# Oral health knowledge in pre-school children: A survey among parents in central Italy

**DOI:** 10.4317/jced.55378

**Published:** 2019-04-01

**Authors:** Francesca Calcagnile, Daniela Pietrunti, Nicola Pranno, Gianni Di Giorgio, Livia Ottolenghi, Iole Vozza

**Affiliations:** 1 DDS, PhDing. Department of Oral and Maxillo-facial Sciences, Sapienza University of Rome; 2 RDH. Department of Oral and Maxillo-facial Sciences, Sapienza University of Rome; 3 DDS, PhD. Department of Oral and Maxillo-facial Sciences, Sapienza University of Rome; 4 DDS. Department of Oral and Maxillo-facial Sciences, Sapienza University of Rome

## Abstract

**Background:**

The aim of this survey was to evaluate the knowledge and awareness of parents about potential oral health risk factors and correct management of oral hygiene of their preschool children.

**Material and Methods:**

The participation to the survey was proposed to all parents of 3-5 year aged children attending a kindergarten in Campobasso. A self-administered questionnaire was completed to obtain information regarding demographic and education variables, knowledge about caries and its transmission, infant feeding practice, maternal oral health during pregnancy, parents and children’s oral hygiene habits and risk behaviors (e.g., sharing cutlery, tasting of baby food, nightly using of baby bottles or pacifier), oral health prevention and role of school.

**Results:**

Overall, the parents of 101 children consented to fill the questionnaire. Data analysis showed that only 24% of respondents was aware of the potential vertical transmissibility of cariogenic bacteria through contaminated saliva. It is still a common trend from 61% of parents tasting food of their child. On 101 children, 30% used pacifier and 17% used baby bottle with milk during night and among these children 41% for more than 2 years. Parents reported no toothbrushing for 57% of the children in their first 3 years of life.

**Conclusions:**

From this survey, independently on parents education, it emerges as still nowadays parents are not fully trained and informed about the management of their child’s oral hygiene and as it’s necessary a parental oral health promoting program to control children oral health risk status, starting from school.

** Key words:**Oral health, pre-school children, dental caries, oral prevention.

## Introduction

The incidence of dental diseases, especially caries, remains high in pediatric age, despite the undoubted improvements obtained in terms of general health (1). In the USA, caries is found in a percentage of about 30% of children between 2 and 5 years of age (2), and in many Countries represents the most common chronical childhood disease (3) . Furthermore, there is a large difference in dental caries development between 1-3 year- aged children: in Japan, in fact, where dental examinations are performed at the age of 18 months and 3 years, it was found that the prevalence of dental caries increased almost ten times when the children turned from 1.5 to 3 years of age (4). Prevention and early intervention are very important because pediatric patients affected by caries may experience pain and often subsequent sleeping disorders, altered eating habits (5), reduced speech production, body weight loss and growth decrease (6). Furthermore, being affected by caries in early childhood means definitely developing it successively (7).

Understanding the factors that influence oral health behaviors it is important for the development of strategies to prevent dental caries and promote oral health, as identified by the WHO (8). Therefore, to promote preventive interventions appears important to understand the social value that parents and communities ascribe to primary teeth (9), and then it’s required the synergistic interaction of all the professionals responsible for maintaining and restoring oral health.

The pediatrician plays a fundamental role as guarantor and responsible for the health of subjects in the developmental age.

In accordance with the prevalence criteria of the diseases and the relevance of the health problem that they represent, particular attention has been paid to the following topics.

- prevention of carious lesions;

- prevention of gingivitis;

- prevention of oral mucosa diseases;

- prevention of orthopedic jaw problems;

Parents have a fundamental role in helping the child to adopt lifestyles that are favorable to health and well-being. In fact, parents respond to the needs of their children by providing them with care and protection. So the aim of this survey was to evaluate the knowledge and awareness of parents and caregivers about potential oral health risk factors and correct management of oral hygiene of their 3-5-year aged children.

## Material and Methods

The study protocol conformed to the ethical guidelines of the 1975 Declaration of Helsinki was approved by the appropriate ethics committee of Sapienza University of Rome (n.4681). The study took place in a kindergarten called “Colozza” Institute in Campobasso (CB). Anonymous questionnaires were administered to all parents of 155 children aged between 3 and 5 years, attending the kindergarten. The managerial staff and the parents of the children, have been made aware of the purpose of the study, and they signed their informed consent. The study began on 01st June 2017 and ended on 30th September 2017. The questionnaire validation was checked in a previous study (10). The questionnaire administered was described in  Table 1. The data gathered with the questionnaire were recorded with a specially designed computer program and collated in a Microsoft Excel database. Descriptive statistics were computed for each items and the percentage of participants answers to each item was calculated. The analysis of the data was performed using SPSS 14.0 for Windows (SPSS Inc., Chicago, IL, USA).

**Table 1:**
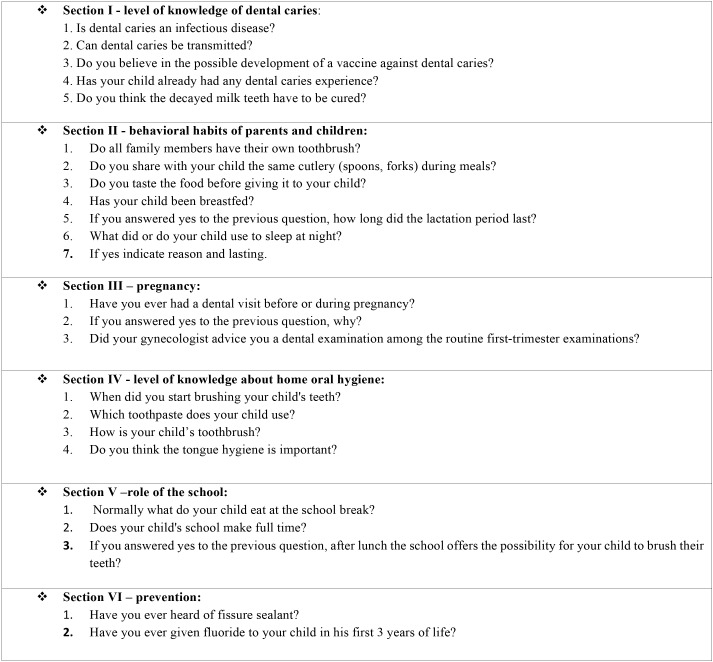
The administered questionnaire.

## Results

For this survey, 155 questionnaires were administered; of these, 101 questionnaires were returned completed. So the study involved a sample of 101 children including 45 boys and 56 girls. Among the parents who contributed to the completion of the questionnaire, 53 were at their first experience as a parent and the remaining 48 were found to have more than one child. On 101 respondents, 82 were mothers (45 graduates and 37 having high school diploma) and 19 were fathers (13 graduates and 6 having high school diploma).

The results are reported subdivided according to the previously mentioned sections and summarized in  Table 2 and  Table 3.

**Table 2:**
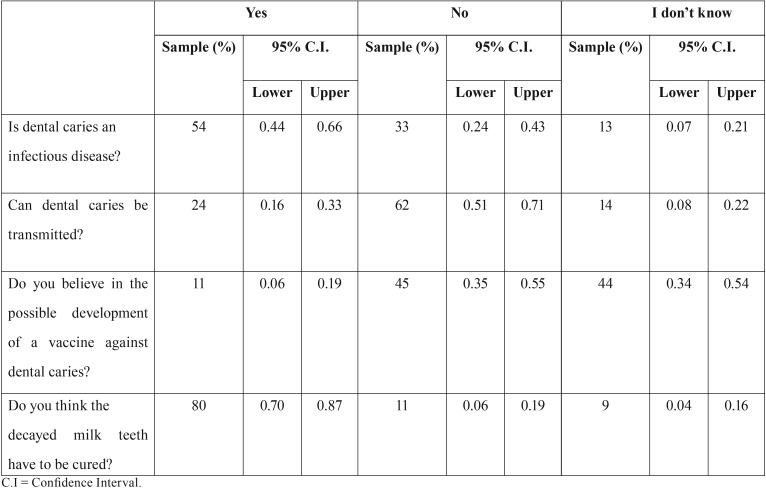
Level of knowledge of dental caries.

**Table 3:**
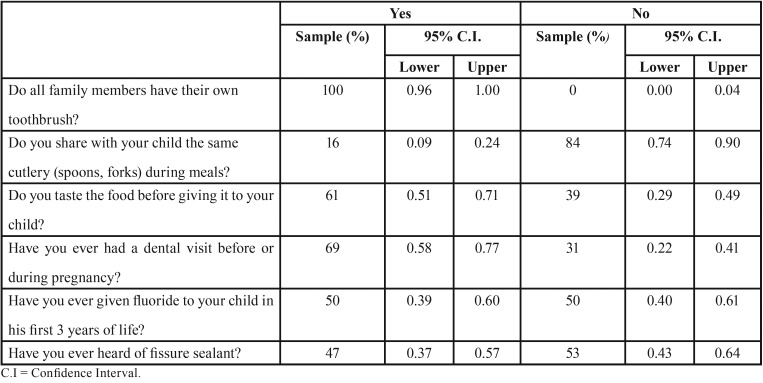
Behavioral habits of parents.

-Section I - Level of knowledge of dental caries

The results are reported in  Table 2. On 101 parents surveyed, only 55 (54%, 95% CI [0.44, 0.64]) recognized dental caries as an infectious disease. These data make us understand how information about the origin and causes of dental caries are yet unknown to many people nowadays. Even more significant data are recorded on the transmission of dental caries: only 24 (24%, 95% CI [0.16, 0.33]) answered affirmatively. This confirms lack of knowledge from too many parents about bacterial flora is cause of caries and possible transmission to the child through saliva since the first days of life: during the pregnancy and breastfeeding is therefore important to make sure you have a healthy mouth (11,12). About the question concerning a possible development of a vaccine against dental caries, the 11 parents who answered correctly (11%, 95% CI [0.06, 0.19]) confirm the scarce knowledge of the topic. The cause of these results is surely due to a misinformation by the scientific institutions. Despite these first not very encouraging data, from the study carried out, it turned out that only 12 children (12%, 95% CI [0.06, 0.2]) have already had some experience of dental caries, compared to 88 (88%, 95% CI [0.88, 0.94]) of healthy children. Among this 12%, 10 children (83%, 95% CI [0.52, 0.98]) received dental care while 2 (17%, 95% CI [0.02, 0.48]) did not.

-Section II - Behavioral habits of parents and children

**Figure 1:**
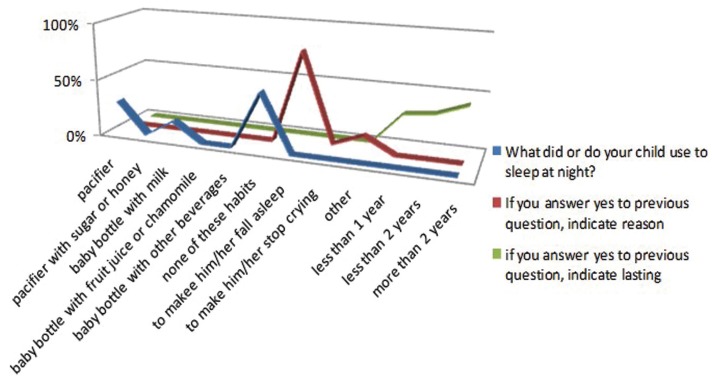
Behavioral habits of children.

Despite the shortcomings that still exist among the parents regarding the oral health status of their child, all of the parents examined, it is certain and sure that all members of the family must have their own home oral hygiene. The concept of transmission of dental caries from the parent to the child remains less clear. In fact, still 62 parents (61%, 95% CI [0.51, 0.71]) taste food of their child. On 101 surveyed children, 30 (30%, 95% CI [0.21, 0.40]) used pacifier and 17 (17%, 95% CI [0.10, 0.26]) used baby bottle with milk during night and among these children 7 (41%, 95% CI [0.18, 0.67]) for more than 2 years (Fig. 1).

-Section III – Pregnancy

Given the possible increase in oral diseases during pregnancy, due to normal physiological changes related to gestation (13), it was considered appropriate to include in the questionnaire also questions regarding the pregnancy of the mothers of the children examined for the study. In this regard, it was found that 70 mothers (69%, 95% CI [0.58, 0.77]) underwent a dental visit before or during pregnancy. Of this 69%, it emerged that 25 performed a dental examination for check-up, 23 carried out a scaling, 14 for pain and 8 for dental caries. In addition, of this 69%, only 4 mothers (5%, 95% CI [0.02, 0.14]) said that it was their gynecologist who recommended a dental visit between routine examinations of the first trimester of pregnancy, 66 (95%, 95% CI [0.86, 0.98]) was not recommended by their gynecologist. From the data collected, it is clear that still today there is a not sufficient information from the gynecologists about prevention of oral cavity diseases of the pregnant patient, despite literature shows the importance of collaboration between them and dentists and oral hygienists in order to improve prevention strategies for oral health of pregnant women (14,16).

-Section IV - Level of knowledge abouthome oral hygiene

**Figure 2:**
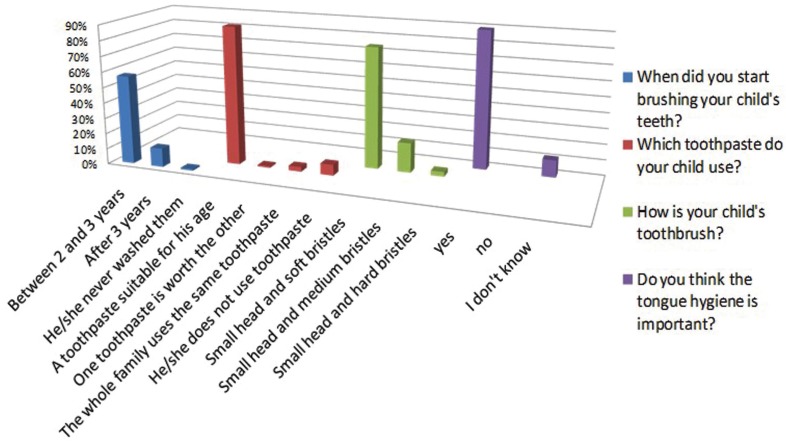
Level of knowledge about home oral hygiene.

Regarding the toothbrushing action, 58 (57%, 95% CI [0.47, 0.67]) said they started between 2 and 3 years of age, as reported in Fig. 2. Regarding home oral hygiene, 90 (89%, 95% CI [0.81, 0.94]) of parents who collaborated in the survey, reported that their children use daily toothpaste suitable for his age, and unfortunately still 7 (7%, 95% CI [0.03, 0.14]) of the children in the sample do not use toothpaste. About the characteristics of a toothbrush for children, 79 (78%, 95% CI [0.69, 0.86]) of parents answered correctly, stating that child’s toothbrush must have a small head and soft bristles. Surely more positive data in this part of the questionnaire have been found regarding the importance of tongue hygiene. In fact, 90 (89%, 95% CI [0.81, 0.94]) of parents answered affirmatively and correctly.

-Section V –The role of the school

Interesting and in some ways also questionable data, have been found in this part of the questionnaire. Regarding the feeding of children during the school break, 42 (42%, 95% CI [0.32, 0.52]) of parents declared that their children eat a sandwich, still 37 (37%, 95% CI [0.27, 0.47]) snack and fruit juice, only 7 (7%, 95% CI [0.03, 0.14]) fresh fruit and 14 (14%, 95% CI [0.08, 0.22]) yogurt. On 101 children, 35 (35%, 95% CI [0.26, 0.45]) has full time, compared to 65 (65%, 95% CI [0.55, 0.75]) who take part in teaching activities only in the morning. Of this 35%, only 4 parents (11%, 95% CI [0.03, 0.27]) stated that the school offers the opportunity for their child to brush their teeth after lunch (perhaps for special needs) against unfortunately 89% (95% CI [0.73, 0.97]) to which this possibility does not is offered. These data, which, despite the information that is daily disseminated through the “media” and the development of prevention programs to prevent diseases affecting the oral cavity from an early age, leave us disconcerted. The school therefore, in light of the analysis of these data, is certainly to be considered one of the main responsible in not promoting of the prevention of oral health of children.

-Section VI – Prevention

In the context of pediatric oral health prevention, the data collected are not yet fully exhaustive. In the recent years, in fact, many steps have been taken forward, but there is still a long way to go. As reported on  Table 3, 54 (53%, 95% CI [0.43, 0.64]) of parents are unaware about fissure sealing, despite this technique of prevention of dental caries, has been known for several years(17).

## Discussion

The results obtained by this survey brought to our attention four points on which it is still necessary to intervene incisively. The first point concern the caries transmission. The mother plays a role that is now recognized as fundamental in the oral health of her child, being her closest to the child: Manchanda *et al.* (18) had demonstrated that educating mothers in oral health led to a reduction of dental caries development in children. The habit of sharing food and cutlery (horizontal transmission) expose infants at increased risk for the development of caries if daily at-home oral health hygiene practices are lacking (19). The best thing to do is to improve parents’ oral hygiene as much as possible to reduce the presence of micro-organisms responsible for dental caries, with the result of better protecting the child. The second point concerns the children nightly habits: the wrong use of baby bottle and pacifier.

The results that emerged from our study on this point confirm that still too many children (with a total percentage of 49%) continue to use or have used them for a long period of time (41% more than 2 years).These data are rather alarming, both due to the high risk of the onset of childhood caries (ECC / baby caries syndrome) (20)and to the high risk of compromising the optimal balance and development of the dental and skull - facial complex of the child. Even today it’s not clear if bottle feeding is more cariogenic than breastfeeding: although some studies have not found an association between breastfeeding and dental caries (21,23), other authors have reported the existence of such an association (24). At the same time some studies stated bottle feeding represents a risk factor for dental caries (25-27), although another author did not find such an association (28). A systematic review published in 2015 stated that on the bases of the current scientific evidence breastfeeding can be considered to have greater protective effect against dental caries than bottle feeding (29). The survey analyzes another point: oral hygiene at home. Our study found that the majority of parents in the sample (57%) started brushing their children’s teeth , for the first time, between 2 and 3 years and only 30% after the eruption of first tooth.

In view of these results, it is clear that independently on parents education, still nowadays parents are not fully trained and informed about the management of their child’s oral hygiene. It was demonstrated that daily tooth brushing, also supervised by the parents, decreased the risk of caries development (30). About the prevention it’s important also to inform parents about the use of fluoride. This has been recommended for the prevention of dental caries by the Centers for Disease Control and Prevention (31,32) because of its role as inhibitor of demineralization of enamel and also for remineralizing enamel crystals. Furthermore, the presence of fluoride inhibits the acid production of pathogenic bacteria (33,34). If children begin to approach the toothbrush, toothpaste and the figure of the dentist and / or dental hygienist early, oral hygiene will become a consolidated habit that will lead to adulthood reducing the occurrence of diseases of the oral cavity, as defined by the Italian -Ministry of Health guidelines on oral hygiene (35). In this context, school plays a fundamental role (36).

According to the current bureaucracy, it depends on school director’ s discretion to allow young pupils to brush their teeth or not. In this regard, in fact, it emerged that on 101 children, 35% had a full time having lunch at school, and among them, school offer the opportunity to dedicate time to oral hygiene only to 11 % (probably for specific reasons or upon delivery of a medical certificate). Primary prevention health promotion projects should be therefore organized targeting them in order to improve:

- during pregnancy reducing cariogenic bacteria levels prior to infant tooth eruption in an effort to avoid the transmission to their babies. Future mothers can be informed by social networks and/or professionals in order to support them in identifying and minimizing risk behaviors affecting their children;

- preventive dental visit at young age in order to avoid or reduce future oral diseases;

- preventive oral hygiene programs at school; if School educates children, its program should also include oral hygiene education. However, although in our study educational or socioeconomic level have not been statistically correlated and this constitutes a limitation of the study, parents of lower educational or socio-economic level have major barriers of access to oral health educational information from a cultural point of view as confirmed by Literature (37,38). So further research is advised in this field to better understand factors involved in the pathway to pre-school children oral health promotion.
